# Vitamin D Status in Migraine Patients: A Case-Control Study

**DOI:** 10.1155/2014/514782

**Published:** 2014-01-02

**Authors:** Alireza Zandifar, Samaneh sadat Masjedi, Mahboobeh Banihashemi, Fatemeh Asgari, Navid Manouchehri, Homa Ebrahimi, Faraidoon Haghdoost, Mohammad Saadatnia

**Affiliations:** ^1^Physiology Research Center, Department of Physiology, Isfahan University of Medical Sciences, Isfahan 81745-319, Iran; ^2^Medical Student Research Center, Isfahan University of Medical Sciences, Isfahan 81745-319, Iran; ^3^Medical Student Research Center, Faculty of Medicine, Islamic Azad University, NajafAbad Branch, NajafAbad 8514143131, Iran; ^4^Department of Neurology and Isfahan Neurosciences Research Center, Isfahan University of Medical Sciences, Hezarjarib Avenue, Isfahan 81745-319, Iran

## Abstract

*Background*. There have been few studies on the relation between vitamin D and migraine. We investigated the prevalence of vitamin D deficiency in migraine patients and compared it with a control group. We also evaluated the relationship of vitamin D deficiency with severity of migraine. *Methods*. 105 newly diagnosed migraine patients and 110 controls, matched for age, sex, socioeconomic status, education, and sun exposure, were enrolled during the spring of 2011. 25-Hydroxy vitamin D [25(OH)D] plasma levels were measured by chemiluminescence immunoassay. *Results*. The mean ± SE concentration of 25(OH)D was 13.55 ± 0.91 ng/mL in cases and 13.19 ± 1.19 ng/mL in controls. There was no significant difference in 25(OH)D concentration between cases and controls. We found no relationship between severity of headache and 25(OH)D status. *Conclusions*. We did not find any association between migraine and vitamin D status; also, severity of headaches was not related to 25(OH)D level. Further studies with larger sample sizes are required to confirm our results.

## 1. Introduction

Vitamin D deficiency is a global public health issue. Prevalence of vitamin D deficiency is about 30 to 50% in normal populations especially in young women [1, 2]. Despite high sun exposure in middle eastern countries, these countries are among the areas with high prevalence of vitamin D deficiency in the world [3]. Vitamin D is not only important in mineral homeostasis but it is also an anti-inflammatory hormone that can regulate immune responses, cell proliferation, and endothelial function [4, 5]. Also, vitamin D has a negative effect on proliferation of mast cells and can stimulate nitric oxide (NO) [6] (a vasoactive substance that interacts with blood vessels).

With these mechanisms, vitamin D has a potent role in cardiovascular disease [7]. Growing but conflicting evidence has shown a possible relationship between vitamin D and chronic or recurrent painful conditions such as migraine [8].

Migraine is a common hereditary chronic neurovascular disorder, characterized by dysfunction of the autonomic nervous system. In some patients, it has been accompanied by nausea and vomiting with phonophobia, photophobia, and osmophobia [9]. Migraine afflicts 18% of women and 6% of men and has a peak incidence in ages between 25 and 55 [10]. There is growing evidence for a role of endothelial dysfunction in migraine pathophysiology which suggests that migraine is attributed to vascular smooth muscle dysfunction [11].

Hypersensitivity of migraine patients' arteries to vasoactive substance such as NO can be a cause of dysfunction [12]. A number of studies have suggested that migraine attacks increase in cold seasons and higher latitude which may be related to lower vitamin D serum level [13].

One study reported that 40% of migraine patients had vitamin D deficiency [14]. Two case-report studies have shown that treatment with vitamin D and calcium in patients suffering from migraine dramatically reduced frequency and severity of migraine [15, 16]. Improvement of headache symptoms in eight vitamin D insufficient patients that had osteomalacia and tension-type headache (TTH) symptoms has been reported after they took vitamin D and calcium supplements for a short period of time [17]. In molecular level, Motaghi et al. have shown that vitamin D receptor polymorphisms are associated with migraine without aura and also headache severity [18].

However, there are no studies that compare vitamin D levels between control group and migraine patients with a large enough sample size and adjustments for age and sex. Also, there are no studies that compare the frequency and severity of headaches based on the vitamin D status. Therefore, we investigated prevalence of vitamin D deficiency in migraine patients and compared it with a control group. We also evaluated vitamin D deficiency in relation to severity of migraine.

## 2. Methods and Materials

### 2.1. Study Design

We conducted a case-control study in the spring of 2011 from April to June. We selected the sample group from patients referred to neurology clinic in Alzahra hospital in Isfahan, Iran (32_390 north, 51_430 East, and 1475 m above sea).

Patients' diagnosis was based on ICHD-II diagnostic criteria [19]. 73 patients aged between 15 and 65 years, with newly diagnosed migraine, were consecutively enrolled. Use of vitamin D supplements, drugs with known effect on serum concentrations of 25-hydroxy vitamin D [25(OH)D], and specific prophylactic drugs such as Triptans and history of rheumatologic, gastrointestinal, liver, and kidney dysfunctions were defined as exclusion criteria. A control group of 98 people was selected from the patients' family members with no history of migraine that was matched for age, education, sex, sunlight exposure, and socioeconomic status. Informed consent was obtained from the participants before entering the study. The study was approved by the Ethics Committee of Isfahan University of Medical Sciences.

### 2.2. Data Collection and Measurement

Demographic data including age, sex, duration of sun exposure, place of residence, and level of education was obtained from both groups. Patients were evaluated with regard to their headache duration and headache frequency and filled out the migraine severity scale (MIGSEV). The MIGSEV was developed by EL Hasnaoui in 2003; it is a simple severity scale with four items including intensity of pain, disability in daily activities, tolerability, and nausea which categorizes patients in three groups of intensity; mild, moderate, and severe. This instrument is highly reliable, reproducible, and sensitive [20]. Persian translation of MIGSEV has been used in another study as a valid scale [21]. Women were asked about menstrual aggravating effects on headaches. Five mL of venous blood was drawn from each participant and immediately centrifuged. Serum samples were then frozen and kept at minus 70 degrees centigrade. Then, samples were measured for plasma levels of 25-hydroxy vitamin D using a chemiluminescent immunoassay kit (“25 OH Vitamin D total assay, Diasorin Liaison”) by LIAISON analyzer. Based on previous studies and reports of Institute Of Medicine (IOM), we considered serum levels of 25-hydroxy vitamin D of >20, 10–20, and <10 ng/mL as normal, insufficient, and deficient, respectively [22].

### 2.3. Data Analysis

We analyzed our data with the SPSS software (version 18.0, Chicago, IL, USA). Mann-Whitney *U* test was used for the comparison of quantitative variables between two groups and the Kruskal-Wallis test was used to compare mean of 25(OH)D between multiple groups, since our data was not normally distributed. The relationships between vitamin D status (mild, moderate, or severe deficiency) and different categorized variables (age, sex, case-control) were investigated using the Chi-square test and the calculation of odds ratios with 95% confidence intervals (95% CI). The sample size achieved 80% of the statistical power. A two-tailed *P* value of less than 0.05 was considered statistically significant.

## 3. Results

One hundred and five cases and 110 controls that were matched for age, sex, duration of sun exposure, area of residency, and level of education were enrolled in the study. Demographic data are presented in Table 1.

The mean **± **SE concentration of vitamin D was 13.55 ± 0.91 ng/mL in cases and 13.19 ± 1.19 ng/mL in controls. There was no significant difference in the vitamin D concentration between cases and controls. The prevalence of normal, insufficient, and deficient vitamin D levels was 20%, 34.3% and 45.7% in cases and 18.2%, 30% and 51.8% controls, respectively. Comparison between different groups of vitamin D deficiency revealed no relation between the severity of deficiency and the presence of disease.

Prevalence of different features of migraine in patients and vitamin D status in each category is shown in Table 2.

There were no significant relationships between vitamin D concentration and frequency of headaches per month, presence of nausea, positive family history of migraine, duration of headache, and headache aggravation with menstruation. Different states in each MIGSEV items (level of nausea, pain tolerability, intensity of pain, and level of disability) were not associated with significant differences in vitamin D concentrations. Also, there was no significant difference in vitamin D levels between different grades of MIGSEV.

Vitamin D status in different subgroups of sex, age, duration of daily sun exposure, place of residence, and education levels in both cases and controls is demonstrated in Table 3. Within both cases and controls, there was a significant difference between level of 25(OH)D in males and females (*P* = 0.046 and *P* = 0.019, resp.). No differences were found in level of 25(OH)D between cases and controls, when analyses were conducted separately for men and women. Subjects in cases and controls with less sun exposure had significantly lower 25(OH)D levels than subjects with more exposure to sun light (*P* = 0.004 and *P* = 0.000, resp.). There was no significant difference between cases and controls with the same daily sun light exposure. 25(OH)D levels were not significantly different in patients under the age of 25 compared to older patients (*P* > 0.05) and also there was no significant difference between patients under the age of 50 and older patients (*P* > 0.05). There were no significant differences between subjects from rural and urban areas in the level of 25(OH)D.

## 4. Discussion

Based on our results, there was no significant difference in 25(OH)D plasma levels between cases and controls. Our analysis also did not show any relationship between 25(OH)D plasma levels and the severity of headaches among patients.

Vitamin D deficiency has been defined based on various references. In most of the studies, 25(OH)D plasma levels lower than 10 ng/mg are considered deficient [22], but defining the threshold for the levels of vitamin D insufficiency is a controversial issue [23, 24]. IOM recommended the levels of 20 ng/mL (50 nmol/liter) covering the requirements of at least 97.5% of the population and this critical level will be useful for clinicians for patients' management [22]. Also, most of the studies reported that high majority of complications related to low levels of vitamin D such as hypoparathyroidism, bone fractures, and multiple sclerosis are becoming at levels below 20 ng/mL [25]. In contrast, several references suggested the 25(OH)D plasma levels of 30 ng/mL as an optimal level of vitamin D [25]. Mithal et al. reported that the percentage of vitamin D insufficiency is high or very high in most European countries particularly elderly patients dependent on considering the required serum 25 (OH)D level, either 20 or 30 ng/mL, although, in some regions, such as South Asia and Middle East, vitamin D deficiency is very common in all age groups, from neonates to the elderly. For instance up to 70% of adolescent girls in Iran had 25(OH)D levels below 25 nmol/L, consistent with findings in Saudi Arabia [24]. It looks that the 30 ng/mL level of vitamin D serum as a critical cut-point of defining vitamin D insufficiency would not be applicable to all regions in the world as there are other variables that affect the vitamin D status in different populations including mean age of the population, air pollution, nutritional status, socioeconomic status, regional latitude, and dietary vitamin D fortification [26].

Finally, it seems that prevalence of vitamin D inadequacy has been overestimated by considering inappropriate cut-points such as serum 25 (OH)D above 30 ng/mL as a normal level of vitamin D [22]. So, in the present study, we defined sufficient, insufficient, and deficient as 25(OH)D levels of more than 20, 10–20, and less than 10 ng/mL, respectively.

The vitamin D plasma level can be measured by different laboratory methods including chemiluminescence assay, radioimmunoassay (RIA), and enzyme-linked immunosorbent assay (ELISA). Different methods can lead to significantly different reported levels of vitamin D in the same sample population. Intermethod variability of the 25(OH)D plasma levels can impact on further analysis of data from different studies. Data standardization is of great importance when different methods are used [27].

To our knowledge, this is the first case-control study that evaluates the status of vitamin D in patients with migraine headache. We did not find any significant difference in 25(OH)D plasma levels between cases and controls. A summary of studies on the relationship between vitamin D and headache (including design, sample size, type of headache, severity of headache assessment, mean vitamin D, and vitamin D measurement method) is shown in Table 4.

In a topical review, Straube et al. claimed that high quality evidence had not found any convincing relationship between chronic pain and vitamin D status [8]. Kjaergaard et al. in a cross-sectional study with a large sample concluded that vitamin D is not related to migraine. The latter, however, is limited as it has used a questionnaire to identify patients rather than identifying them clinically. Questionnaires may miss the patients or overdiagnose people with migraine. Also, in this study, the relationship between severity and frequency of headaches and vitamin D is not evaluated [28]. Our results did not show any relation between vitamin D and either severity of the headaches or their frequency.

Some studies have suggested that higher latitudes are associated with more sever and frequent headaches and therefore can originate from lower 25(OH)D plasma levels due to diminished sun exposure. This conclusion, however, is not totally reliable as there are regions with lower latitudes and worse vitamin D status. It can be assumed that vitamin D insufficiency may be caused by a multiple factors and latitude alone may not be responsible for lower 25(OH)D plasma levels. Seasonal changes in 25(OH)D plasma levels are reported in the literature [8].

Thys-Jacobs reported dramatic reduction in frequency and duration of headaches after supplemental vitamin D administration. However, these results were from small case reports in two premenopausal and two postmenopausal women without control group [15, 16]. Despite these results we did not find any relation between 25(OH)D plasma levels and menstrual related aggravation of headaches. The therapeutic effect of vitamin D supplementation is thought to be due to higher absorption of magnesium; however, these treatments were accompanied by calcium supplements also and the results could be attributed to both vitamin D and calcium or even the synergetic effect of the treatment.

Wheeler suggested in his study that migraine and vitamin D could be associated as 40.7% of the patients in that study had vitamin D deficiency. The study is limited though, as the diagnostic criteria for migraine were not clear, the study did not have a matched control group and patients who received vitamin D supplements were not excluded [14]. O'Brien et al. also found a high prevalence of vitamin D deficiency among pediatric patients with recurrent headaches in their study [29]. In another study, Krusz et al. established that there were no significant differences in vitamin D levels between migraine patients and other patients with any other pain disorders [30]; however, the major limitation of these two studies was the lack of a control group.

Prakesh et al. reported positive effects of vitamin D and calcium supplements in tension type headache (TTH) patients. Beneficial outcomes of optimal 25(OH)D levels for TTH patients are also suggested by Keargaard et al. Prakash et al. reported high levels of parathyroid hormone (PTH) in migraine patients which could be due to secondary hyperparathyroidism that was treated by optimal levels of vitamin D supplement. The role of endothelial dysfunction and also the role of nitric oxide (NO) through vasodilatation as triggers for migraine headaches have been well established [11, 12]. There are also studies, claiming that there are higher levels of parathyroid hormone in the systemic circulation of patients with heart failure, which is associated with endothelial dysfunction [31]. Also, it is stated that PTH upregulates the activity of the endothelial nitric oxide synthase eNOS system through protein kinase pathways [32]. On the other hand, vitamin D deficiency can cause secondary hyperparathyroidism. However, whether or not the rise of PTH in this phenomenon contributes to worse migraine headaches through NO release and endothelial dysfunction is a matter of debate that needs further study.

Knutsen et al. stated that vitamin D status has a much stronger relationship with headache rather than either musculoskeletal pain or fatigue [33]. Our results did not reveal any relationship between the severity of the vitamin D deficiency and severity of the headaches. Also, 25(OH)D plasma levels were not different between MIGSEV items (nausea, intensity of pain, pain tolerability, and disability).

We found that, in both cases and controls, female patients had significantly lower levels of 25(OH)D, but there was no difference in subjects of the same gender between cases and controls. Higher vitamin D deficiency and more severe pain in females have been reported in previous studies as well [8, 33].

The relationship between age and severity of vitamin D deficiency was established by Knutsen et al. [33]. We found no relation between the age and levels of 25(OH)D. Also, according to our results, vitamin D level was not related to level of education and place of living (rural or urban).

The prevalence of vitamin D deficiency is roughly similar between cases and controls and between matching age groups. This suggests a high prevalence of vitamin D deficiency in both healthy population and migraine patients which implies a common underlying cause. It seems appropriate to conduct population-based observational studies to better assess the role of vitamin D in incidence, severity, and treatment of migraine headaches [13, 17].

There are some limitations to our study. Our criteria only included new cases of migraine headache; although this benefited the study since new migraine patients had no history of medication, it also led to a small sample size. Lack of body mass index (BMI) matching between cases and controls was another weakness of our study; however, we matched the patients and controls for sun exposure and subjects who received vitamin D supplements were excluded.

In conclusion, we did not find any association between migraine and vitamin D status; also, severity of headaches was not related to vitamin D level. Other studies with larger sample sizes and randomized double-blind clinical trials should be performed to confirm this hypothesis.

## Figures and Tables

**Table 1:** Characteristics of case and control.

Characteristic	Cases	Controls
Sample size (*n*)	105	110
Age (mean ± SD)	33.59 ± 0.97	32.46 ± 0.91
Age groups		
<30.5 years	50 (47.6%)	58 (52.7%)
≥30.5 years	55 (52.4%)	52 (47.3%)
Sex		
Female	80 (76.2%)	89 (80.9%)
Male	25 (23.8%)	21 (19.1%)
Place of residence		
Urban	52 (49.5%)	58 (52.7%)
Rural	53 (50.5%)	52 (47.3%)
Level of education		
Below high school diploma	44 (41.9%)	44 (40.0%)
Above high school diploma	61 (58.1%)	66 (60.0%)
Duration of sun light exposure (mean ± SE)	98.14 ± 6.54 min/day	94.51 ± 8.93 min/day
Groups of sun light exposure		
0–120 min/day	83 (79.0%)	90 (81.8%)
≥120 min/day	22 (21.0%)	20 (18.2%)

**Table 2:** Prevalence of different features of migraine in patients and vitamin D status as sufficient level (more than 20 ng/mL) and deficient level (less than 10 ng/mL) in each category.

Item (Case)	Vitamin D level (ng/mL)		*P* value
	Less than 10	More than 20	
Frequency of headache per month			
≤15	41 (78.8%)	17 (81.0%)	0.840
>15	11 (21.2%)	4 (19.0%)	
Nausea			
Yes	40 (76.9%)	14 (66.7%)	0.366
No	12 (23.1%)	7 (33.3%)	
Family history			
Positive	38 (73.1%)	14 (66.7%)	0.584
Negative	14 (26.9%)	7 (33.3%)	
Menstrual effect			
Yes	22 (45.8%)	11 (73.3%)	0.063
No	26 (54.2%)	4 (26.7%)	
Intensity of pain			
Mild	0 (0.0%)	0 (0.0%)	0.109
Moderate	13 (25.0%)	5 (23.8%)	
Intense	26 (50.0%)	15 (71.4%)	
Very Intense	13 (25.0%)	1 (4.8%)	
Level of nausea			
None	12 (23.1%)	7 (33.3%)	0.148
Mild	25 (48.1%)	4 (19.0%)	
Intense	8 (15.4%)	5 (23.8%)	
Vomiting	7 (13.5%)	5 (23.8%)	
Level of disability			
None	5 (9.6%)	2 (9.5%)	0.395
Mild	24 (46.2%)	10 (47.6%)	
Marked	10 (19.2%)	1 (4.8%)	
Confined to bed	13 (25.0%)	8 (38.1%)	
Level of tolerability			
Tolerable	11 (21.2%)	4 (19.0%)	0.946
Barely tolerable	28 (53.8%)	11 (52.4%)	
Intolerable	13 (25.0%)	6 (28.6%)	
MIGSEV total			
Low	6 (11.6%)	4 (19.0%)	0.686
Intermediate	23 (44.2%)	8 (38.1%)	
High	23 (44.2%)	9 (42.9%)	
Duration of headache			
4–12 h	24 (46.2%)	12 (57.1%)	0.695
12–24 h	16 (30.8%)	5 (23.8%)	
24–72 h	12 (23.1%)	4 (19.0%)	

**Table 3:** Vitamin D status in different subgroups in both cases and controls.

Item	Mean of serum vitamin D ± SD (control)	*P* value (within case groups)	Mean of serum vitamin D ± SD (case)	*P* value (within control groups)	*P* value (between case and control groups)
Sex					
Female	11.84 ± 1.34	0.019	12.52 ± 1.13	0.046	0.702
Male	18.91 ± 2.19		16.82 ± 1.15		0.381
Residency					
Rural	14.97 ± 2.04	0.159	15.12 ± 1.19	0.084	0.949
Urban	11.60 ± 1.30		11.94 ± 1.37		0.857
Level of education					
Below high school diploma	13.00 ± 1.68	0.895	13.08 ± 1.26	0.672	0.967
Above high school diploma	13.32 ± 1.64		13.88 ± 1.29		0.793
Duration of sun exposure					
0–120 min/day	10.67 ± 0.81	0.000	12.20 ± 1.01	0.004	0.238
≥120 min/day	24.53 ± 4.74		18.60 ± 1.81		0.234
Group age					
≤25	13.21 ± 1.88	0.993	12.21 ± 1.95	0.457	0.722
>25	13.18 ± 1.49		13.90 ± 1.04		0.693
≤50	12.85 ± 1.21	0.307	13.24 ± 0.95	0.243	0.803
>50	17.56 ± 5.56		17.30 ± 3.26		0.968

The Mann-Whitney test was used to compare mean of vitamin D in different groups.

**Table 4:** Summary of the literature on relation between vitamin D and headache.

The first author (year)	Study design	Patient sample size	Type of headache	Headache severity assessment	Methods of vitamin D level assessment	Mean of vitamin D level in patient (ng/mL)	Effect/association of vitamin D (found by authors)
Kjaergaard et al. [28] (2012)	Cross-sectional	4061	Migraine (*N* = 322) Nonmigraine headache (*N* = 3739)	Yes	Electrochemiluminescent immunometric assay	24.62 (migraine patient) 24.48 (nonmigraine headache)	Not associated
Knutsen et al. [33] (2010)	Cross-sectional	63	Patients with chief complaint of headache	Yes	High-pressure liquid chromatography mass spectrometry (HPLC-MS)	14	Associated
O'Brien et al. [29] (2010)	Cross-sectional	300	Episodic migraine (*N* = 173) Chronic migraine (*N* = 127)	No	Not reported	24.19 (episodic migraine) 23.19 (chronic migraine)	Associated
Krusz et al. [30] (2010)	Cross-sectional	100	Migraine/ headache (*N* = 34) pain syndromes (*N* = 33)	No	Not reported	29.3 (migraine/ headache) 28.2 (pain syndromes)	Not associated
Wheeler [14] (2008)	Cross-sectional	54	Chronic migraine	No	Not reported	34.6	Associated
Prakash [17] (2009)	Case report	8	Chronic tension-type headache	No	Not reported	7	Effective
Thys-Jacobs [15] (November 1994)	Case report	2	Migraine	Yes	Not reported	Not reported	Effective
Thys-Jacobs [16] (October 1994)	Case report	2	Menstrually-related migraines	Yes	Not reported	Not reported	Effective
